# Interfractional shifts of the carina and hilum during radiotherapy for non‐small cell lung cancer: A retrospective study to identify associated factors

**DOI:** 10.1002/acm2.70382

**Published:** 2025-11-21

**Authors:** Kazuhito Ueki, Mitsuhiro Nakamura, Norio Araki

**Affiliations:** ^1^ Department of Radiation Oncology National Hospital Organization Kyoto Medical Center Kyoto Japan; ^2^ Department of Advanced Medical Physics Graduate School of Medicine Kyoto University Kyoto Japan

**Keywords:** anatomic landmarks, cone‐beam computed tomography, lymph nodes, mon‐small cell lung cancer, radiotherapy, tumor burden

## Abstract

**Background:**

Thoracic lymph nodes are critical targets in radiotherapy for locally advanced non‐small cell lung cancer (LA‐NSCLC), but their accurate localization is complicated by interfractional shifts.

**Purpose:**

To evaluate the interfractional carina and hilum shifts as surrogates for thoracic lymph node areas during conventional fractionated radiotherapy for LA‐NSCLC and to explore baseline characteristics of patients related to these shifts.

**Methods:**

For 23 patients, the carina and hilum nearest the primary tumor were marked on daily cone‐beam computed tomography (CBCT) images after vertebrae‐based registration. The interfractional shifts of these two points were determined based on comparison of the first and subsequent CBCT scans. The patients were grouped using dynamic time warping clustering, and their baseline characteristics were compared. Population margins were calculated using the van Herk formula. Shift directionality was analyzed using principal component analysis.

**Results:**

The proportion of shifts of >5 mm in all CBCT scans was 10.9% for the carina and 22.8% for the hilum. The patients were grouped into small‐shift, medium‐shift, and large‐shift groups based on their hilum shifts. The large‐shift group had the largest shifts, with a median (interquartile range) of 3.9 mm (2.4–5.2) for the carina and 6.3 mm (4.4–10.1) for the hilum. The gross tumor volume (GTV) within 2 cm of the central airways (proximal GTV) was significantly different among groups (*p* < 0.01). Greater proximal GTVs were correlated with large median shifts of each patient (correlation coefficients: carina, 0.57; hilum, 0.76) and showed a tendency toward unidirectional hilum shift. Population margins reached 4.7–7.5 mm for the carina and 6.9–13.1 mm for the hilum in the highest proximal GTV tertile (median 30.6 cm^3^).

**Conclusions:**

Variations were observed in the shifts across patients and lymph node positions. Greater proximal GTVs were correlated with large and directional hilum shifts, indicating the potential benefits of tailoring adaptive radiotherapy strategies.

## 1 INTRODUCTION

Thoracic lymph nodes are critical targets for definitive radiotherapy in locally advanced non‐small cell lung cancer (LA‐NSCLC). Despite advances in treatment modalities, regional lymph node recurrence rates remain high at 40%–50%, and more than half of the cases involve in‐field recurrence.^1^, ^2^ To improve control rates, precise targeting of metastatic lymph nodes is crucial. However, interfractional shifts of the thoracic anatomical structures are the main challenges encountered during conventional fractionated radiotherapy administered over 6 weeks.^3^ These changes, primarily due to tumor regression, occur at rates of approximately 35%–60%^4^, ^5^ and may lead to discrepancies between the planned and delivered doses. Moreover, thoracic lymph nodes are close to the heart and esophagus, and anatomical shifts can increase adverse effects.^6^ This impedes dose escalation for lymph node metastases.

Cone‐beam computed tomography (CBCT) imaging has become a key tool for managing anatomical shifts.^7^, ^8^ However, its low contrast limits its usefulness for visualizing thoracic lymph nodes.^9^, ^10^ In contrast, standard CT modalities provide clear images, but reliable identification of the hilar nodes without contrast‐enhanced CT remains difficult. Consequently, previous studies on interfractional shifts in lymph nodes have primarily focused on mediastinal lymph nodes.^11^, ^12^

To overcome these limitations, we used daily CBCT scans combined with anatomical landmarks to evaluate interfractional shifts within the key lymph node areas during definitive radiotherapy for LA‐NSCLC. This approach allows for continuous monitoring and quantitative assessment of three‐dimensional shifts in the thoracic anatomy. Furthermore, using common surrogate points enables a consistent comparison of shifts across patients. This helps to identify patterns related to patient backgrounds and to recognize patients who have substantial shifts.

## 2 MATERIALS AND METHODS

### 2.1 Patients

This retrospective study included patients with LA‐NSCLC who received definitive radiotherapy with conventional fractionation (2 Gy per fraction for a total dose of 60–70 Gy) between April 2020 and March 2024 at our institution. The eligibility criteria were pathological confirmation, no history of thoracic surgery or radiotherapy, and image‐guided radiation therapy using daily CBCT. To concentrate on quantifiable predictors of anatomical shifts, we excluded patients with significant baseline abnormalities that often necessitated unpredictable replanning. These abnormalities included atelectasis, extensive pleural effusion, and pneumothorax.^13^, ^14^, ^15^ We included patients without lymph node metastasis because this study aimed to evaluate the anatomical shifts of the lymph node areas rather than the lymph nodes involved. Twenty‐three patients met the above criteria. Table 1 presents the baseline characteristics of these patients.

**TABLE 1:** Baseline patient characteristics.

Characteristic	Overall, *n* = 23	
Age, median (range), year	75	(43, 88)
Sex (%)		
Male/Female	18/5	(78.3/21.7)
Location of primary tumor (%)		
RU/RM/RL/LU/LL/M	5/0/8/6/4/0	(21.7/0/34.8/26.1/17.4/0)
Stage (%)		
IIIA/IIIB/IIIC	14/8/1	(60.9/34.8/4.3)
Histology (%)		
Ad/Sq/NSCLC	7/12/4	(30.4/52.2/17.4)
Total GTV, median (range), cm^3^	65.3	(11.2, 297.5)
Radiotherapy dose (%)		
60 Gy/70 Gy	19/4	(82.6/17.4)
Concurrent chemotherapy (%)		
Yes/No	19/4	(82.6/17.4)

Abbreviations: Ad, adenocarcinoma; GTV, gross tumor volume; LL, left lower; LU, left upper; M, mediastinum; NSCLC, non‐small cell lung cancer; RL, right lower; RM, right middle; RU, right upper; Sq, squamous cell carcinoma.

The institutional review board approved this study and waived the need for informed consent due to the retrospective design of the study.

### 2.2 Treatment planning

Patients underwent free‐breathing four‐dimensional (4D) planning CT (pCT) (Aquilion LB, Canon Medical Systems, Ohtawara, Japan) with 3‐mm slices and were immobilized on an individualized vacuum pillow. Using the Eclipse treatment planning system (version 15.1; Varian Medical Systems, Palo Alto, USA), gross tumor volumes (GTVs) of the primary tumor and/or metastatic lymph nodes were delineated on the maximum intensity projection, accounting for respiratory tumor motion.^16^ The GTV was expanded isotropically by 5 mm to create the clinical target volume, with an additional 5‐mm margin added to generate the planning target volume (PTV). The treatment was planned with 10 MV volumetric modulated arc therapy on average 4D‐CT, with 50% of the PTV receiving the prescribed dose.

### 2.3 Treatment

CBCT images were acquired using an on‐board imager system (Varian Medical Systems) to correct the patient setup. Each CBCT image set, captured during free breathing, comprised axial slices with 2‐mm spacing over a 16‐cm field of view. The CBCT images were rigidly registered to the pCT based on the bony anatomy, and three‐dimensional (3D) translational shifts were applied to maintain the tumor within the PTV. Offline adaptive replanning was used for systematic deviations or misalignments.

### 2.4 Retrospective CBCT registration

CBCT scans from the first to the 30th fraction were rigidly registered to the pCT using six degrees of freedom (translation and rotation) based on the vertebra. For patients who received >30 fractions, the analysis was limited to scans up to the 30th fraction.

### 2.5 Surrogate points of lymph node areas

To quantify the interfractional shifts in lymph node areas using CBCT, we adopted the approach proposed by Hoffmann et al.,^17^ which uses anatomical landmarks instead of directly assessing the metastatic lymph nodes. In the lung window setting, two anatomical landmarks were selected for daily CBCT. The carina, located at the superior tip of the cartilaginous ridge between the main bronchi, was selected as a surrogate for the mediastinum. For the hilum, the entrance of the lobar bronchus, closest to the primary tumor, served as the surrogate point and was adjusted based on the location of each tumor in each patient to track shifts related to tumor regression. To minimize interobserver variability, a single board‐certified radiation oncologist defined the points of interest (POIs).

### 2.6 Interfractional shifts of surrogate points and patient group

For the two POIs on bony registered CBCT, the interfractional shifts from the first to subsequent fractions were recorded in three directions: left‐right (LR), anterior‐posterior (AP), and superior–inferior (SI). The resulting three dimensional distances were calculated using the square root of the sum of squared shifts in these directions.

To cluster patients without predefined labels, we used a dynamic time warping (DTW) algorithm with barycenter averaging.^18^, ^19^ DTW measures the distance between two time series by aligning them, with data points adjusted in time to minimize the differences. Barycenter averaging iteratively refines an initial average time series, reduces the DTW distance between the average and individual time series, and robustly captures the common pattern within each cluster. We decided to cluster the patients into three distinct groups based on hilum shifts after visualizing the interfractional shifts in both the carina and hilum.

### 2.7 Systematic/random shifts and resulting margins estimated at the population level

Systematic shifts were calculated as the standard deviation of the mean shifts of the patients, and random shifts were calculated as the root‐mean‐square of the standard deviation of shifts of each patient; both were determined per group.^20^ These values were used to calculate theoretical margins covering interfractional shifts at the population level, using the formula developed by van Herk et al.^21^

### 2.8 Predictive factors of categorized groups

To investigate factors affecting the interfractional shift trends, we analyzed the differences between the baseline characteristics of the groups. The variables encompassed both clinical and quantitative information derived from pCT. The clinical variables included age, sex, tumor location, clinical stage, histology, and use of concurrent chemotherapy. Because of the limited number of participants, we regrouped the categorical variables into two: histology (squamous cell carcinoma vs. non‐squamous cell carcinoma) and stage (IIIA vs. IIIB/IIIC). The quantitative variables included not only the total GTV (primary and nodal) but also spatial parameters assessing the proximity of the tumor to the central anatomical structures. These parameters included the 3D distance from the carina to the center of the total GTV on pCT.^22^ Additionally, we defined “proximal GTV” as any GTV located within 2 cm of the proximal bronchial tree (distal 2 cm to the trachea, carina, and main bronchi).^23^

### 2.9 Directionality of interfractional shifts

We used principal component analysis (PCA) to determine whether the shifts in the POIs during radiotherapy were uniformly distributed across all directions or showed preferential directional tendencies. PCA was applied to the spatial coordinates (LR, AP, and SI) of each patient and recorded for all fractions. This analysis transformed these coordinates into three new orthogonal axes: principal component 1 (PC1), PC2, and PC3. PC1 explained the greatest variance, followed by PC2 and PC3. The explained variance ratios (EVRs) of PC1, PC2, and PC3, which were summed up to 100% for each patient, quantified the contribution of each principal direction to the overall shifts.

### 2.10 Statistical analysis

The Kruskal–Wallis test was used to compare the continuous data of the three independent groups, whereas Fisher's exact test was used to compare the categorical data. Dunn's test was used for post‐hoc pairwise comparisons of continuous data. Bonferroni correction was applied to adjust the *p*‐values for multiple comparisons. The Wilcoxon signed‐rank test was used to assess the differences between the matched continuous variables. Spearman's correlation coefficient was used to explore relationships between continuous data; a simple linear regression with its 95% confidence interval and coefficient of determination (*R* ^2^) was additionally calculated as a descriptive reference. Two‐sided *p*‐values of <0.05 denoted statistical significance. All analyses were performed using R software (version 4.3.3; www.r‐project.org).

## 3 RESULTS

### 3.1 Overview of interfractional shifts and patient groups

**FIGURE 1:**
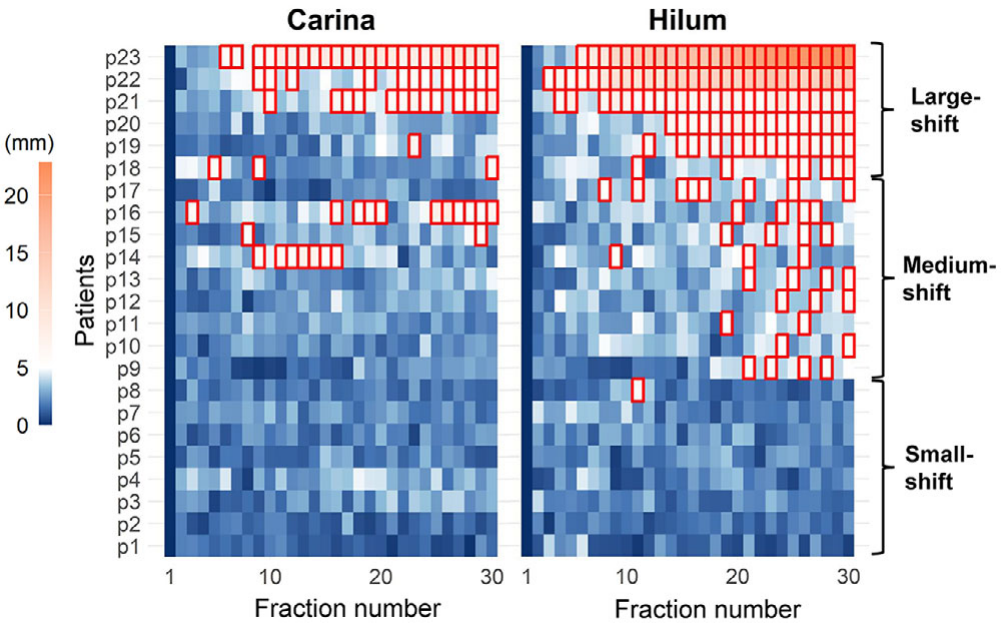
Heatmap of the interfractional shifts of the carina and hilum for all patients. The deep blue color indicates no shift from the first cone‐beam computed tomography. As the shifts approach the standard 5‐mm planning target volume margin, the color transitions from blue to light red and intensifies to a deep red when exceeding 5 mm. Each heatmap cell represents one fraction, and cells with red borders indicate shifts exceeding 5 mm. The labels small‐shift, medium‐shift, and large‐shift on the right side of the figure indicate the results of patient grouping.

Figure 1 illustrates the comprehensive time trend of shifts in the two POIs across all patients. The proportion of interfractional shifts of >5 mm in 667 CBCT scans (23 patients, with shifts analyzed from fractions 2 to 30) was 10.9% for the carina and 22.8% for the hilum. The patients were divided into three groups, as shown in Figure 1. Patients 1–8 consistently had low variability in the hilum shifts, with shifts remaining below 5 mm during later fractions of radiotherapy (hereinafter referred to as the small‐shift group, *n* = 8). Patients 9–17 often had hilum shifts exceeding 5 mm during later fractions of radiotherapy (the medium‐shift group, *n* = 9). Patients 18–23 experienced substantial shifts exceeding 5 mm early during radiotherapy, and these shifts continued to exceed 5 mm until the end of radiotherapy (the large‐shift group, *n* = 6).

### 3.2 Differences in interfractional shifts among groups

During radiotherapy, the median 3D carina shifts (interquartile range) across all fractions were 1.8 mm (1.3–2.6), 2.5 mm (1.9–3.5), and 3.9 mm (2.4–5.2) for the small‐shift, medium‐shift, and large‐shift groups, respectively (*p* < 0.0001). Similarly, the median 3D hilum shifts were 1.8 mm (1.2–3.6), 3.5 mm (2.5–4.4), and 6.3 mm (4.4–10.1) for the small‐shift, medium‐shift, and large‐shift groups, respectively (*p* < 0.0001). The hilum shifts were larger than the carina shifts in the medium‐shift and large‐shift groups (*p* < 0.0001); however, no significant difference was observed in the small‐shift group (*p* = 0.48).

### 3.3 Systematic/random shifts and resulting margins at the population level, stratified by shift magnitude

**FIGURE 2:**
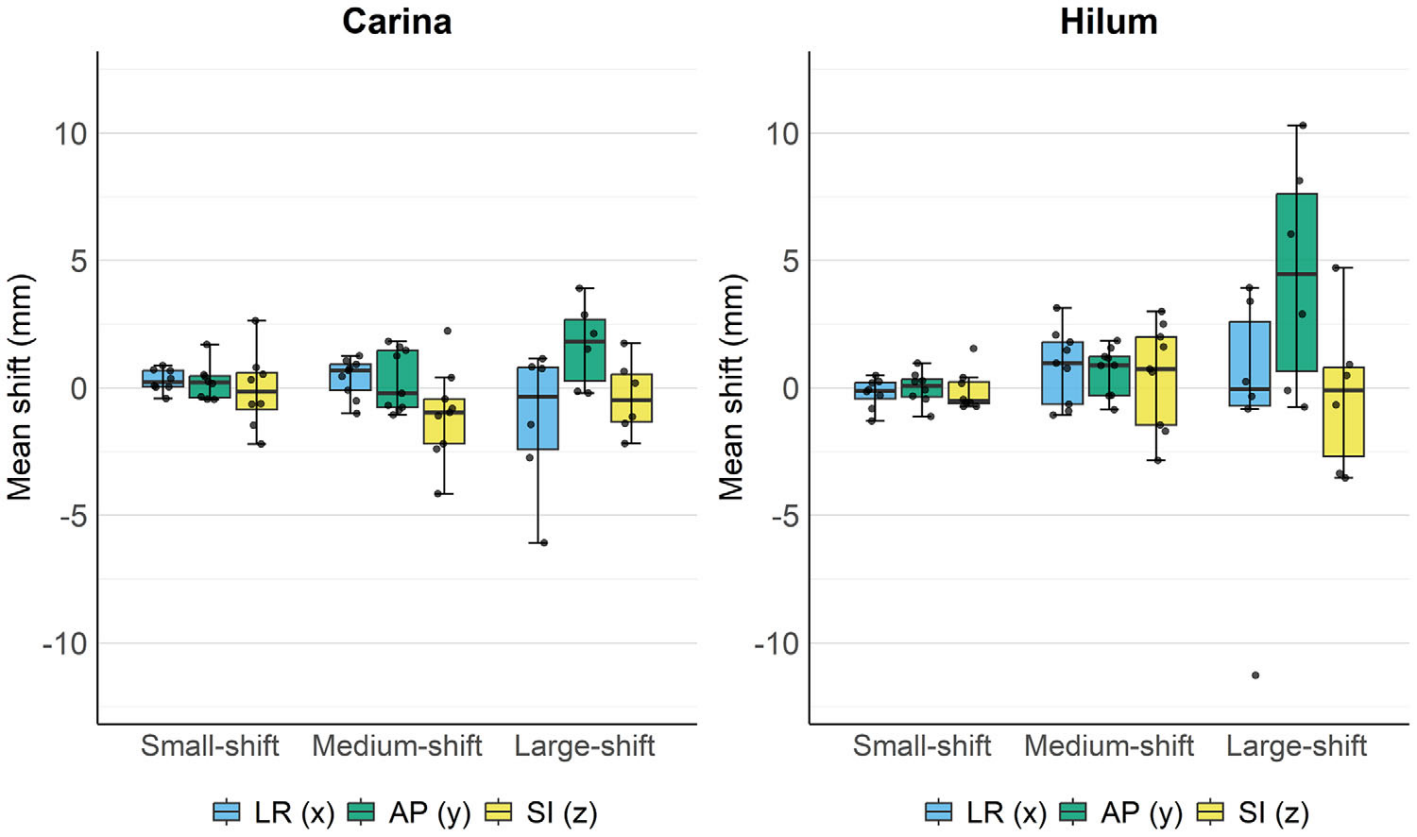
Distribution of patient‐level mean shifts stratified by shift‐magnitude groups and directions. Box plots of patient‐level mean shifts stratified by shift‐magnitude groups (small, medium, large) and directions (left‐right [LR], anterior–posterior [AP], superior–inferior [SI]). Positive values denote shifts toward the patient's left, posterior, and superior. Boxes indicate the interquartile range (IQR) with the median line; whiskers extend to 1.5× the IQR; dots represent individual patients (jittered).

Figure 2 presents the distribution of patient‐level mean (systematic) shifts stratified by shift‐magnitude groups for the carina and the hilum. For both sites, the small‐shift group showed patient‐level mean shifts centered near 0 mm across all directions with narrow inter‐patient spread (interquartile range). In the medium‐shift group, dispersion increased relative to the small‐shift group. In the large‐shift group, the hilum showed a greater median shift in the positive AP direction (toward anterior) and the widest inter‐patient spread. In the LR direction of this group, both the carina and hilum had medians close to 0 mm but exhibited a wide range of values, which for the hilum was due to inclusion of outliers. These distributions provide the basis for estimating systematic shifts at the population level. The systematic and random shifts and resulting margins for both the carina and hilum stratified by the small‐shift, medium‐shift, and large‐shift groups, are summarized in Table 3. The systematic shift and margin for the hilum were notably larger for the large‐shift group than for the other groups; they measured 3.1–5.5 and 9.1–15.8 mm, respectively. Regarding the carina, the margin required to cover the shifts was approximately 5 mm, except for the LR direction in the large‐shift group. In the small‐shift and medium‐shift groups, the shifts for both the carina and hilum were the largest in the SI direction among the three directions.

### 3.4 Differences in patient baseline characteristics among groups

**FIGURE 3:**
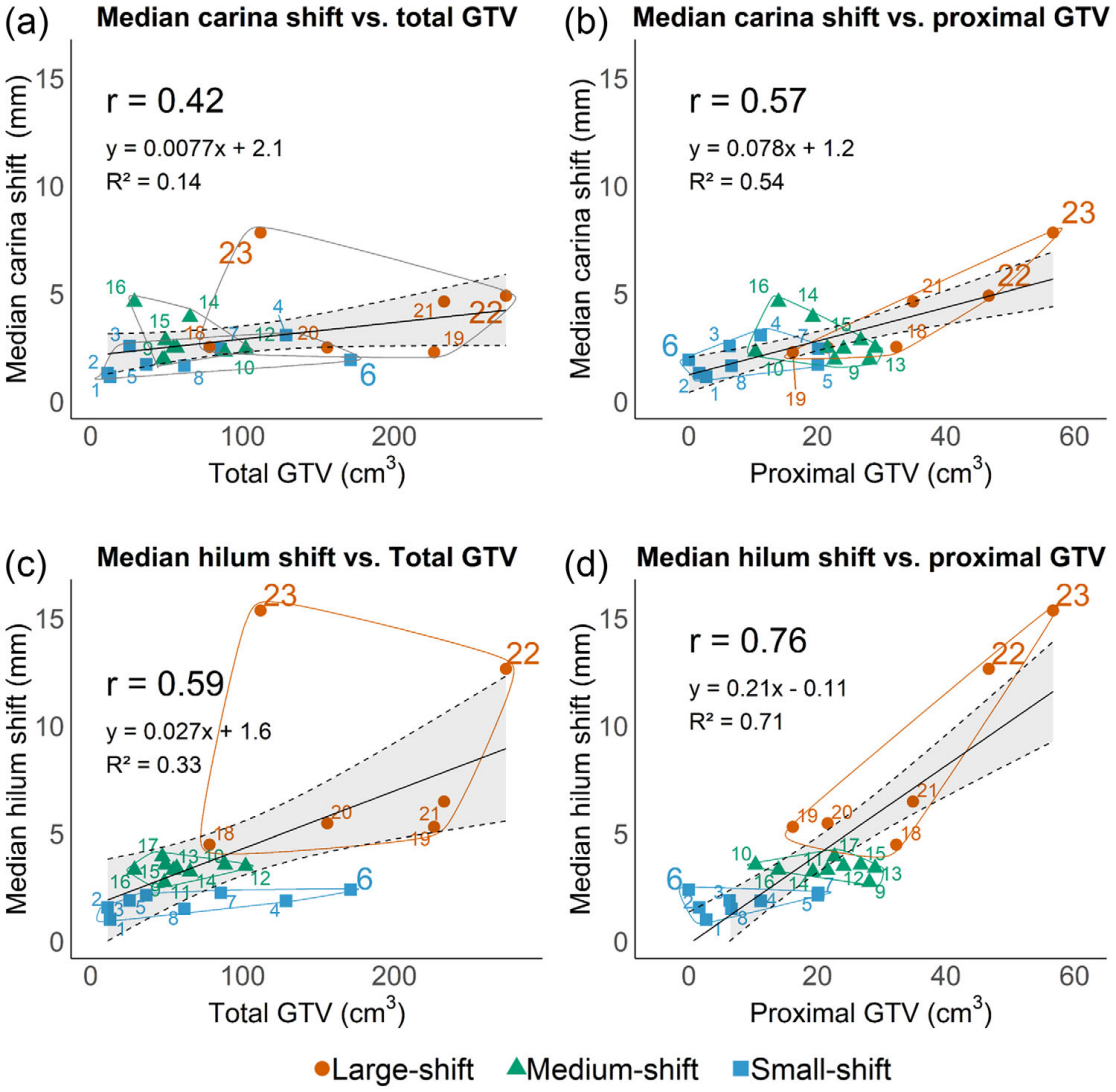
Relationships between median shift values during radiotherapy and tumor volumes. Each patient's data point is marked by a number corresponding to the identifiers in Figure 1. Spearman's correlation coefficients (*r*) indicate associations. A linear regression line (solid) is shown with its 95% confidence interval (dashed) for the fitted mean, together with the coefficient of determination (*R* ^2^) as a reference. The median carina shifts are compared with (a) the total gross tumor volume (GTV) and (b) the proximal GTV. The median hilum shifts are compared with (c) the total GTV and (d) the proximal GTV. Data points are grouped into ellipses according to the groups.

**FIGURE 4:**
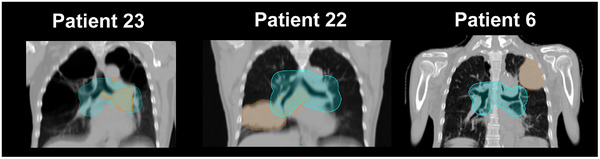
Representative planning computed tomography (CT) images of selected patients. Planning CT images of Patients 23, 22, and 6 are shown in comparable coronal planes. Areas shaded in orange represent the total gross tumor volume (GTV), and those in light blue represent the region within 2 cm of the proximal bronchial tree. The regions where orange and light blue overlap delineate the proximal GTV. Images from three representative patients are shown, displayed in comparable coronal planes.

Among the baseline characteristics, significant differences in the total (*p* < 0.05) and proximal (*p* < 0.01) GTVs were observed among the three groups (Table 2). To examine whether a large tumor volume was correlated with large shifts, we examined the correlations between the median 3D shift for each patient during radiotherapy and the total or proximal GTV. For the carina, the proximal GTV showed a stronger correlation with the median shift than the total GTV (correlation coefficient: 0.57 and 0.42, respectively) (Figure 3a,b). For the hilum, the proximal GTV demonstrated a stronger correlation with the median shift than the total GTV (correlation coefficient: 0.76 and 0.59, respectively) (Figure 3c,d). Examination of individual cases helped further elucidate these correlations (Figure 4). For instance, Patient 23 had the largest proximal GTV (56.7 cm^3^) and median hilum shift (15.4 mm) despite having only the seventh largest total GTV (111.8 cm^3^). Fifty‐one percent of the tumor of this patient was located within 2 cm of the proximal bronchial tree, which likely contributed to the large shifts. Patient 22 showed a median hilum shift of 12.6 mm. This patient had the largest total GTV (297.5 cm^3^), but the proximal GTV (46.7 cm^3^) was smaller than that of Patient 23, resulting in smaller median shifts of both the carina and hilum. In contrast, Patient 6 had a peripheral tumor with the fourth largest total GTV (171.2 cm^3^) but no proximal GTV, resulting in shifts of <5 mm throughout the radiotherapy.

**TABLE 2:** Differences in the baseline characteristics among the three groups.

Characteristic	Small‐shift *n* = 8	Medium‐shift *n* = 9	Large‐shift *n* = 6	*p*‐value
Age, year	76 (60, 82)	75 (47, 80)	67 (43, 88)	0.47
Sex (%)				0.24
Female	3 (37.5)	2 (22.2)	0 (0.0)	
Male	5 (62.5)	7 (77.8)	6 (100.0)	
Primary site (%)				0.41
Left lobe	5 (62.5)	3 (33.3)	2 (33.3)	
Right lobe	3 (37.5)	6 (66.7)	4 (66.7)	
Primary site (%)				0.093
Lower lobe	2 (25.0)	5 (55.6)	5 (83.3)	
Upper lobe	6 (75.0)	4 (44.4)	1 (16.7)	
Stage (%)				0.58
IIIA	6 (75.0)	5 (55.6)	3 (50.0)	
IIIB/IIIC	2 (25.0)	4 (44.4)	3 (50.0)	
Histology (%)				0.97
Non‐Sq	4 (50.0)	4 (44.4)	3 (50.0)	
Sq	4 (50.0)	5 (55.6)	3 (50.0)	
Carina to center of total GTV, cm	4.1 (2.7, 6.2)	2.7 (1.2, 4.7)	4.1 (1.7, 5.4)	0.13
Total GTV, cm^3^	49.2 (11.2, 171.2)	54.5 (28.7, 101.9)	190.9 (78.2, 297.5)	**0.011**
Proximal GTV, cm^3^	6.5 (0, 20.1)	22.7 (10.4, 29.0)	33.6 (16.2, 56.7)	**0.002**
Concurrent chemotherapy (%)				0.75
Yes	6 (75.0)	8 (88.9)	5 (83.3)	
No	2 (25.0)	1 (11.1)	1 (16.7)	

*Note*: All continuous data are presented as median (range).

Abbreviations: 3D, three‐dimensional; GTV, gross tumor volume; Sq, squamous cell carcinoma;

### 3.5 Systematic/random shifts and resulting margins at the population level, stratified by proximal GTV

The exploratory analysis identified proximal GTV as a key factor associated with the interfractional shift magnitude; we subsequently stratified patients by proximal GTV to calculate population‐level margins. Because no established cut‐off value exists, we divided patients into tertiles of proximal GTV. Patients were stratified into tertiles of proximal GTV, with cut‐off values at 14.7 and 23.6 cm^3^ (33rd and 67th percentiles, respectively). This yielded three groups with median (range) volumes of 6.5 cm^3^ (0–13.9), 20.1 cm^3^ (16.2–22.7), and 30.6 cm^3^ (24.1–56.7) for the first (*n* = 7), second (*n* = 8), and third tertiles (*n* = 8), respectively. The population‐level systematic and random shifts and resulting margins per axis for both the carina and hilum are summarized in Table 4. For both the carina and hilum, margins in the LR and AP directions increased across proximal GTV tertiles, whereas margins in the SI direction did not show a consistent trend. In the highest proximal GTV tertile (median 30.6 cm^3^, range 24.1–56.7 cm^3^), the carina and hilum population margins were 4.7–7.5 and 6.9–13.1 mm, respectively.

### 3.6 Directionality of shifts

Given the substantial hilum shifts and the margins that covered them in the large‐shift group, we hypothesized that large proximal GTVs were correlated with the directional tendency of hilum shifts. For the carina shifts, the proximal GTV showed a correlation with the EVRs of the PCs (correlation coefficient: 0.2, −0.2, −0.36 for PC1, PC2, and PC3 EVRs, respectively) (Figure 4a–c). For the hilum shifts, the proximal GTV demonstrated a stronger correlation with EVRs for the PCs than the carina shift (correlation coefficient: 0.58, −0.61, −0.57 for PC1, PC2, and PC3 EVRs, respectively) (Figure 4d–f). The positive correlation for PC1 EVR and negative correlation for PC2 and PC3 EVRs indicate that the hilum shifts primarily occur in the direction of the PC1 axis, with a unidirectional tendency.

## 4 DISCUSSION

To the best of our knowledge, this is the first study to quantitatively evaluate the interfractional shifts of both the carina and hilum during radiotherapy for LA‐NSCLC and explore the patient baseline characteristics associated with a high likelihood of large shifts. Our findings highlight that large pretreatment proximal GTVs were correlated with substantial and directional hilum shifts.

Limited studies have focused on the relationship between lymph node positions and their interfractional shifts. In our study, the margin needed to cover the carina shifts was approximately 5 mm, except for the LR direction in the large‐shift group (Table 3). Weiss et al. reported a 3–5 mm margin of the mediastinal lymph nodes in patients without atelectasis,^11^ which is consistent with our findings on carina shifts. In contrast, the hilum shifts were larger than the carina shifts in the medium‐shift and large‐shift groups. This difference is likely because the hilum is sensitive to changes in the lung condition, whereas the mediastinum, which houses the carina, is stabilized by surrounding structures such as the sternum and spine. For patients in the small‐shift and medium‐shift groups, a standard isotropic PTV margin of 5 mm is reasonable. However, a tailored strategy, such as using different margins based on lymph node location, may be beneficial for patients in the large‐shift group, especially when hilar areas are involved.

**TABLE 3:** Systematic and random shifts along with resulting margins at population level, stratified by shift magnitude groups.

	Carina			Hilum		
Shift group	Small	Medium	Large	Small	Medium	Large
Systematic (mm)						
LR	0.4	0.8	2.8	0.6	1.5	5.5
AP	0.7	1.2	1.6	0.6	0.9	4.5
SI	1.5	1.8	1.5	0.8	2.1	3.1
Random (mm)						
LR	0.7	1.0	1.6	1.0	1.5	2.9
AP	0.8	1.1	1.1	1.0	1.3	2.3
SI	1.0	1.3	1.8	1.3	1.6	2.1
Margin (mm)						
LR	1.6	2.6	8.1	2.2	4.7	15.8
AP	2.3	3.8	4.9	2.3	3.2	12.8
SI	4.5	5.4	4.9	2.9	6.3	9.1

Abbreviations: AP, anterior–posterior; GTV, gross tumor volume; LR, left‐right; SI, superior–inferior.

**TABLE 4:** Systematic and random shifts along with resulting margins at population level, stratified by proximal GTV tertiles.

	Carina			Hilum		
Proximal GTV (median [range], cm^3^)	6.5 (0–13.9)	20.1 (16.2–22.7)	30.6 (24.1–56.7)	6.5 (0–13.9)	20.1 (16.2–22.7)	30.6 (24.1–56.7)
Systematic (mm)						
LR	0.5	0.9	2.6	1.1	1.6	4.5
AP	0.7	1.1	1.9	1.0	0.9	4.2
SI	2.0	1.5	1.4	1.0	2.7	2.2
Random (mm)						
LR	0.7	1.0	1.4	1.0	1.5	2.6
AP	0.8	1.2	1.0	0.9	1.4	2.1
SI	1.1	1.2	1.7	1.5	1.5	2.0
Margin (mm)						
LR	1.8	2.9	7.5	3.4	5.1	13.1
AP	2.4	3.5	5.5	3.0	3.1	11.9
SI	5.8	4.6	4.7	3.5	7.7	6.9

*Note*: Cut‐off values for tertiles were 14.7 and 23.6 cm^3^ (33rd and 67th percentiles).

Abbreviations: AP, anterior–posterior; GTV, gross tumor volume; LR, left‐right; SI, superior–inferior.

Several previous studies have reported that qualitative pathoanatomic conditions, such as atelectasis, require replanning.^11^, ^13^, ^14^, ^15^ However, few studies have identified the quantifiable factors that influence the need for replanning. Mushonga et al. found that a large PTV (primary and nodal) was associated with an increased need for replanning for patients with stage 3 NSCLC undergoing definitive radiotherapy.^24^ Similarly, our study showed that a large total GTV was correlated with large shifts (Figure 3). However, a large proximal GTV was more strongly correlated with large shifts in both the hilum and carina than the total GTV. Our findings suggest that large peripheral tumors may not necessarily undergo large shifts in lymph node areas and centrally located tumors with large proximal GTVs tend to induce pronounced shifts.

Most previous studies have reported significant tumor volume changes during 30–50 Gy/15–25 fractions in patients with LA‐NSCLC,^9^ suggesting that this interval is suited for replanning. However, early and substantial lymph node shifts have also been observed. For example, van Elmpt et al. reported that 24% of patients showed displacements exceeding 5 mm by the second week of radiotherapy.^25^ Our study corroborates these findings, with the large‐shift group demonstrating shifts of >5 mm before the 15th fraction (Figure 1). Moreover, larger proximal GTVs showed an association with larger shifts (Figure 3). These patterns demonstrate that interfractional shifts vary in both onset and magnitude, highlighting the limitations of uniform, time‐based thresholds for replanning. Adaptive radiotherapy (ART) provides a strategy to address this variability by improving dose‐delivery precision and widening the therapeutic index. It is implemented primarily through offline and online approaches,^26^ but each approach has constraints. Although offline ART is compatible with conventional workflows, it requires additional imaging and planning, which may result in “chasing” rapid anatomical changes.^27^ In contrast, online ART enables per‐fraction correction but imposes substantial demands on staff, technology, and infrastructure,^28^ limiting its feasibility for routine use in long courses of radiotherapy. In this context, our analysis provides a rationale for stratifying patients based on pre‐treatment anatomical characteristics. Patients predicted to have small shifts, typically associated with smaller proximal GTVs, may be managed with optimized margin alone. By contrast, patients at risk of medium or large shifts, more often linked to larger proximal GTVs, may benefit from hybrid strategies. For example, later‐accumulating shifts can initially be managed with optimized margins and monitoring, with mid‐course offline replanning performed if dosimetric limits are exceeded. Patients demonstrating substantial early shifts are optimal candidates for online ART, where margin prediction can reduce the need for daily full adaptation by supporting trigger‐based selective workflows. Together, these stratified strategies highlight how shift prediction, offline ART, and online ART can be applied in a complementary and resource‐conscious manner, balancing precision with feasibility in clinical practice.

**FIGURE 5:**
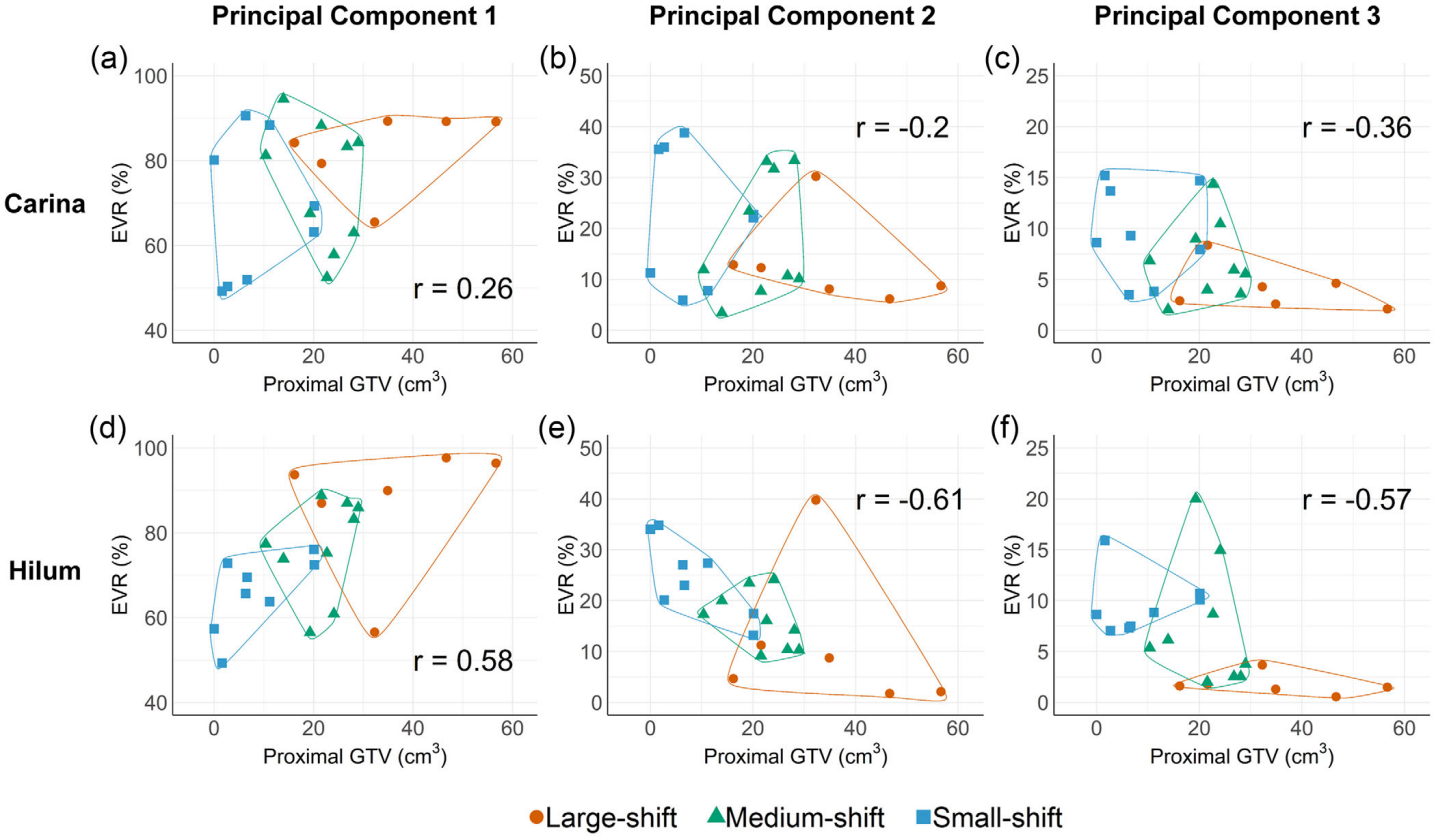
Relationship between proximal gross tumor volumes and explained variance ratios of transformed orthogonal axis. The proximal gross tumor volumes are correlated with the explained variance ratios (EVRs) of principal component 1 (PC1), PC2, and PC3 for (a–c) the carina shifts and (d–f) the hilum shifts. Spearman's correlation coefficients are shown.

For the large‐shift group, population‐based margins of 9.1–15.8 mm may be warranted to cover interfractional shifts, depending on direction (Table 3). However, the application of large margins in all directions results in impractically large PTVs. The ideal approach involves applying margins based on probability levels added to the quadrature.^20^ The methodological concept of quadrature margins, particularly in response to tumor regression during radiotherapy, has not been thoroughly explored. In this study, large proximal GTVs were associated with unidirectional hilum shifts (Figure 5), suggesting that the shifts resulted from the shrinkage of the tumor in the central region. PCA identifies directions of the maximum variance, which is a concept rooted in probability. Using this variance to calculate margins can refine radiotherapy planning for tumors with dynamic shifts. Owing to the limited number of participants, our study did not establish a margin optimization algorithm, highlighting the need for further research.

Our study has some limitations. First, the small sample size and single‐institution, retrospective design limit statistical power and generalizability; thus, the findings should be regarded as exploratory and hypothesis‐generating. In particular, we did not attempt to propose fully individualized margin sizes, because the limited sample and modest correlations precluded robust modeling. Additionally, our results are based on free‐breathing CBCT, which is affected by respiratory motion. Although we defined anatomical landmarks on averaged images without issues, shifts in the small‐shift and medium‐shift groups were large in the SI direction (Table 3). Thus, the observed shifts may reflect tumor shrinkage and uncertainties in the definitions of the POIs due to respiratory motion. Furthermore, we grouped patients autonomously using a data‐driven approach; however, the decision to group them into three based on hilum shifts was arbitrary, suggesting that other groups are viable. Despite these limitations, our findings highlight key factors associated with interfractional shifts and provide a basis for future studies with larger cohorts to establish predictive models and individualized margin recommendations.

## 5 CONCLUSIONS

Considerable variations were observed in the shifts across patients and lymph node positions. Large pretreatment proximal GTVs were correlated with large hilum shifts and associated with their directionality. This suggests the need for ART tailored to patient‐specific characteristics and lymph node positions. This may involve optimizing PTV margins or determining more appropriate replanning timing.

## AUTHOR CONTRIBUTIONS

Kazuhito Ueki designed the study, performed data analysis, and drafted the manuscript. Mitsuhiro Nakamura helped with data analysis. Mitsuhiro Nakamura and Norio Araki reviewed and edited the manuscript. All authors read and approved the final manuscript.

## CONFLICT OF INTEREST STATEMENT

The authors declare no conficts of interest.
